# Complete resolution of generalized annular elastolytic giant cell granuloma with doxycycline^☆^

**DOI:** 10.1016/j.abd.2022.05.011

**Published:** 2023-09-22

**Authors:** Dídac Marín-Piñero, M. Ángeles Sola-Casas, Noelia Perez-Muñoz

**Affiliations:** a Department of Dermatology, Hospital Universitari Sagrat Cor, Grupo Quirónsalud, Barcelona, Spain; b Department of Pathology, Hospital Universitari General de Catalunya, Grupo Quirónsalud, Universitat internacional de Catalunya, Sant Cugat del Vallès, Barcelona, Spain

Dear Editor,

Annular Elastolytic Giant Cell Granuloma (AEGCG) is a rare granulomatous dermatosis with unclear etiology and physiopathology. Histopathological hallmarks are elastolysis and elastophagocytosis. The definitive mechanism that leads to damage of the elastic fibers has not yet been elucidated. Treatment is still challenging and the use of tetracyclines is controversial due to their potential phototoxicity. Herein, we present a case of generalized AEGCG that achieved a complete resolution after daily doxycycline and review the previous cases treated with tetracyclines.

An 88-year-old man with mild hypertension under treatment with lisinopril, visited our dermatology department presenting multiple erythematous and mild pruritic annular plaques with atrophic center located on the back, shoulders, posterior neck, and upper chest that had appeared over the last month (Fig. 1A). Initial differential diagnosis included subacute cutaneous lupus erythematosus, generalized granuloma annulare and annular psoriasis. A punch biopsy taken from an erythematous border showed a scattered dermal granulomatous infiltrate of multinucleated giant cells with elastophagocytosis consistent with the diagnosis of AEGCG (Fig. 2). Laboratory studies including complete blood cell count, glucose levels, liver and renal function, and antinuclear antibodies were normal. Considering that the lesions were not photo-distributed, the little sun exposure by the patient, and the previously reported cases of AEGCG treated with tetracyclines, doxycycline 100 mg daily was initiated. After five months, complete resolution of the lesions and pruritus were observed (Fig. 1B). Nevertheless, the lesions rapidly recurred when doxycycline was discontinued but after its reintroduction, the patient experienced marked improvement during the following 12 months.

**Figure 1:**
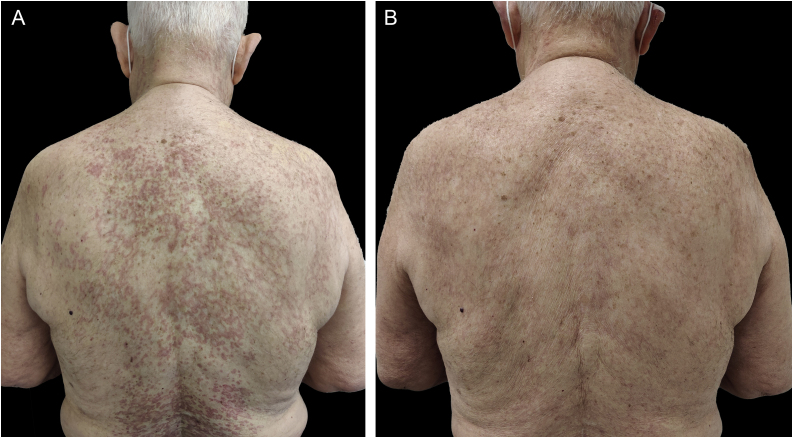
(A) Multiple erythematous annular plaques on the back and posterior neck at the initial visit. (B) Complete resolution of the lesions after 5 months under treatment with doxycycline 100 mg/day

**Figure 2:**
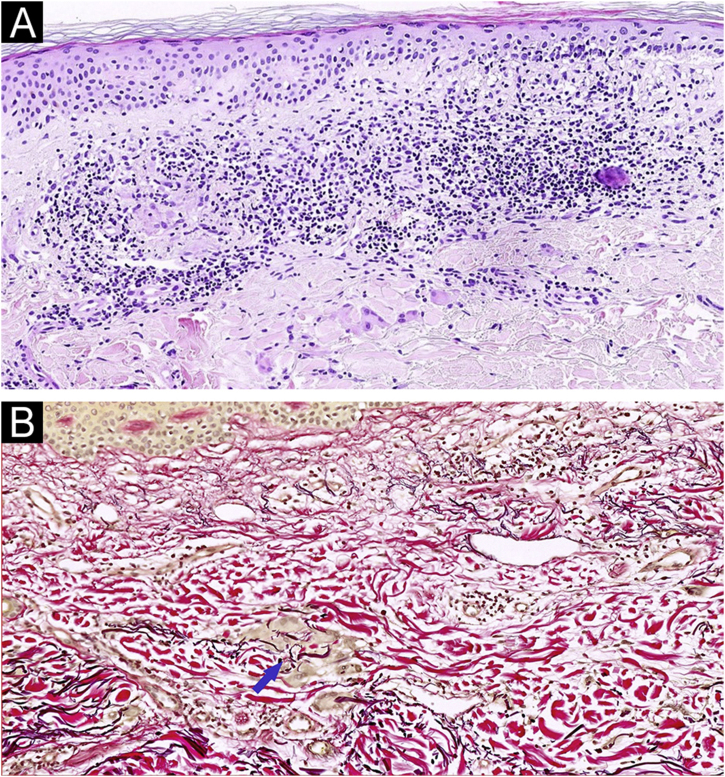
(A) Skin biopsy specimen showing a scattered dermal granulomatous infiltrate with phagocytosis of elastic fibers by multinucleated giant cells. (Hematoxylin & eosin, ×100). (B Elastophagocytosis by multinucleated giant cells demonstrated by elastic fibers stain. The blue arrow indicates the elastophagocytosis. (Verhoeff-Van Gieson; ×100)

AEGCG was first described as actinic granuloma by O’Brien in 1975 to indicate the causal role of actinic damage triggering elastolysis and elastophagocytosis. However, photoaged skin with degenerative elastin changes is rarely associated with elastophagocytosis. Lesions of AEGCG are often located on nonexposed skin so other factors aside from heat and ultraviolet radiation should be considered. Several events may damage the elastic fibers and trigger the inflammatory response that leads to its phagocytosis. One of these triggers may be hyperglycemia in diabetes mellitus, as it has been frequently reported as a concomitant disease. Many other associations such as malignancies and inflammatory diseases have been described but probably the most common presentation is idiopathic.^1^ Despite its benign course, AEGCG can take up to several years to resolve, lesions are usually extensive and may require different treatment modalities to prevent its progression. Anti-inflammatory and anti-granulomatous effects of tetracyclines are well known but they are not regarded as a convenient treatment due to their phototoxicity and the seldom reported risk to trigger AEGCG.^2^ Nevertheless, tetracyclines have shown excellent outcomes with complete responses in both limited^3^, ^4^ and generalized^5^ forms involving sun-exposed and nonexposed skin with no side effects. Table 1^3^, ^4^, ^5^ summarizes the main features of our patient and the three previously published cases of AEGCG treated with tetracyclines.

**Table 1:** Overview of published cases of AEGCG treated with tetracyclines

Article	Cases	Age	Sex	Comorbidities	Pattern and location	Previous treatments	Tetracycline and dose	Outcome
Nanbu et al. (2015)^3^	1	46	Male	None	Solitary annular plaque on the temple	Topical corticosteroid	Minocycline 200 mg/day and 100 mg/day	Resolution after 11 weeks
Kabuto et al. (2017)^5^	1	80	Male	Diabetes mellitus	Annular plaques on the posterior neck, back, chest, upper arms, wrists, dorsum of hands and tights	Topical tacrolimus, topical corticosteroid and oral tranilast	Minocycline 200 mg/day and 100 mg/day	Resolution after 6 months
Jeha et al. (2020)^4^	1	67	Male	Chronic kidney disease, diabetes mellitus and coronary artery disease	Annular plaques on ventral forearms and proximal thighs	Topical and intralesional corticosteroids	Doxycycline 200 mg/day	Resolution after 6 months
Current case	1	88	Male	Hypertension	Annular plaques on the whole back, shoulders, posterior neck, and upper chest	None	Doxycycline 100 mg/day	Resolution after 5 months

In conclusion, tetracyclines should be considered a safe first-line treatment for patients with extensive or generalized AEGCG or a second-line option for localized forms that do not respond to conventional topical treatments.

## Financial support

None declared.

## Authors' contributions

Marín-Piñero D: Approval of the final version of the manuscript; design and planning of the study; drafting and editing of the manuscript; collection, analysis, and interpretation of data; effective participation in research orientation; intellectual participation in the propaedeutic and/or therapeutic conduct of the studied cases; critical review of the literature; critical review of the manuscript.

Sola-Casas MA: Approval of the final version of the manuscript; design and planning of the study; drafting and editing of the manuscript; effective participation in research orientation; intellectual participation in the propaedeutic and/or therapeutic conduct of the studied cases; critical review of the literature; critical review of the manuscript

Perez-Muñoz N: Approval of the final version of the manuscript; design and planning of the study; drafting and editing of the manuscript; effective participation in research orientation; intellectual participation in the propaedeutic and/or therapeutic conduct of the studied cases; critical review of the literature; critical review of the manuscript

## Conflicts of interest

None declared.

## References

[1] Gutiérrez-González E., Pereiro M., Toribio J. (2015). Elastolytic actinic giant cell granuloma. Dermatol Clin..

[2] Lim D.S., Triscott J. (2003). O’Brien’s actinic granuloma in association with prolonged doxycycline phototoxicity. Australas J Dermatol..

[3] Nanbu A., Sugiura K., Kono M., Muro Y., Akiyama M. (2015). Annular elastolytic giant cell granuloma successfully treated with minocycline hydrochloride. Acta Derm Venereol..

[4] Jeha G.M., Luckett K.O., Kole L. (2020). Actinic granuloma responding to doxycycline. JAAD Case Rep..

[5] Kabuto M., Fujimoto N., Tanaka T. (2017). Generalized annular elastolytic giant cell granuloma successfully treated with the long-term use of minocycline hydrochloride. Eur J Dermatol..

